# Robotic spleen-preserving distal pancreatectomy using the first domestic surgical robot platform (the hinotori™ Surgical Robot System): a case report

**DOI:** 10.1186/s40792-024-01808-x

**Published:** 2024-01-18

**Authors:** Kazuki Tomihara, Takao Ide, Kotaro Ito, Tomokazu Tanaka, Hirokazu Noshiro

**Affiliations:** Department of Surgery, Faculty of Medicine, Saga University, 5-1-1 Nabeshima, Saga, 849-8501 Japan

**Keywords:** Spleen-preserving distal pancreatectomy, Robotic surgery, Docking-free system

## Abstract

**Background:**

Robotic pancreatectomy has been performed worldwide mainly using the da Vinci® Surgical System (Intuitive Surgical, Inc., Sunnyvale, CA, USA). Recently, because of the death of some patents related to the da Vinci® system, new surgical robot systems have been introduced that are characterized by unique technical refinements. In Japan, the hinotori™ Surgical Robot System (Medicaroid Corporation, Kobe, Japan) was approved for use in gastroenterological surgery in October 2022. Since then, we have attempted complicated procedures using this robot. In this report, we report our first experience performing spleen-preserving distal pancreatectomy with conservation of the splenic artery and vein using this first Japanese domestic surgical robot.

**Case presentation:**

The patient was a 58-year-old woman with a mass in the pancreatic tail identified during medical screening. Further examinations resulted in a diagnosis of a pancreatic neuroendocrine tumor. The patient consented to surgical resection, and we planned robotic spleen-preserving distal pancreatectomy with conservation of the splenic artery and vein, using the hinotori™. Five trocars, including one port for the assistant surgeon, were placed in the upper abdomen. The operating unit was rolled in from the patient’s right side. The pivot position was set for each robotic arm, and this setting was specific to the hinotori™. The cockpit surgeon performed all surgical procedures, excluding port placement and pancreatic transection. There were no unrecoverable device errors during the operation. The operation time was 531 min, and blood loss was 192 ml. The postoperative course was uneventful. We were able to safely perform this highly complicated surgery for a pancreatic tumor using the first Japanese domestic surgical robot platform.

**Conclusions:**

The first Japanese domestic surgical robot platform, hinotori™, has different features from those of the da Vinci^®^ and performed sufficiently as a surgical robot system in highly advanced pancreatic surgery.

## Background

Pancreatic surgery remains one of the most complicated gastroenterological surgeries because of the nature of the organ [1]. Pancreatic surgery is technically challenging because of the retroperitoneal location of the pancreas and its proximity to the major blood vessels. With advances in technology and surgical techniques, minimally invasive distal pancreatectomy is now considered the standard of care [2]. Robot-assisted distal pancreatectomy is as safe and feasible as open or laparoscopic distal pancreatectomy regarding both the perioperative and long-term oncologic results [3–6].

Although robotic distal pancreatectomy has spread rapidly over the last two decades [7], the procedures have been performed using the da Vinci® Surgical System (Intuitive Surgical, Inc., Sunnyvale, CA, USA) [8]. Recently, companies worldwide have developed new robotic surgical systems characterized by unique technical refinements, because of the death of some patents related to the da Vinci^®^ system [9]. The hinotori™ Surgical Robot System (Medicaroid Corporation, Kobe, Japan) acquired Japanese pharmaceutical approval in August 2020 for use in urology, and the use of this robot was expanded to gastroenterological surgery in October 2022. Immediately thereafter, we used the hinotori™ for robotic distal pancreatectomy with splenectomy [10]. In this report, we discuss our first experience with the more challenging pancreatic surgery, spleen-preserving distal pancreatectomy with conservation of the splenic artery and vein, using the hinotori™.

## Case presentation

**Fig. 1:**
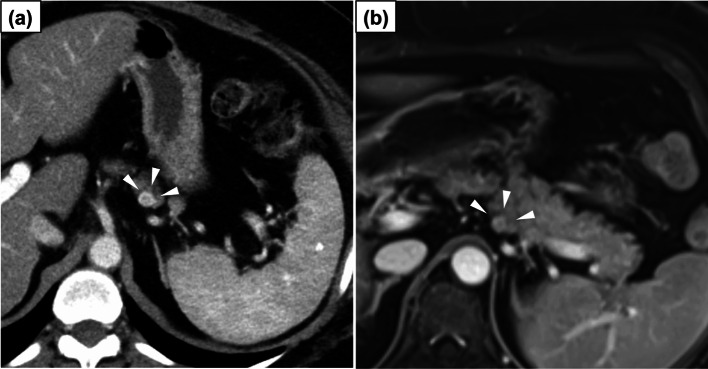
Imaging findings. **a**, **b** Dynamic enhanced computed tomography and magnetic resonance images showing a 13-mm mass in the pancreatic tail with strong contrast enhancement (arrowheads)

A 58-year-old woman underwent a general medical screening and was transferred to our hospital for further evaluation for diabetes mellitus. She had no remarkable medical history, a body mass index of 33.8 kg/m^2^, and glycated hemoglobin level of 11.3%. Dynamic contrast-enhanced computed tomography and magnetic resonance imaging revealed a 13-mm diameter hypervascular lesion in the pancreatic tail (Fig. 1). The tumor was close to the main pancreatic duct. There were no swollen lymph nodes or distant metastases. Endoscopic ultrasonography-assisted fine needle aspiration indicated a G1 neuroendocrine tumor. On the basis of these findings, we planned robotic spleen-preserving distal pancreatectomy with conservation of the splenic artery and vein, using the hinotori™.

**Fig. 2:**
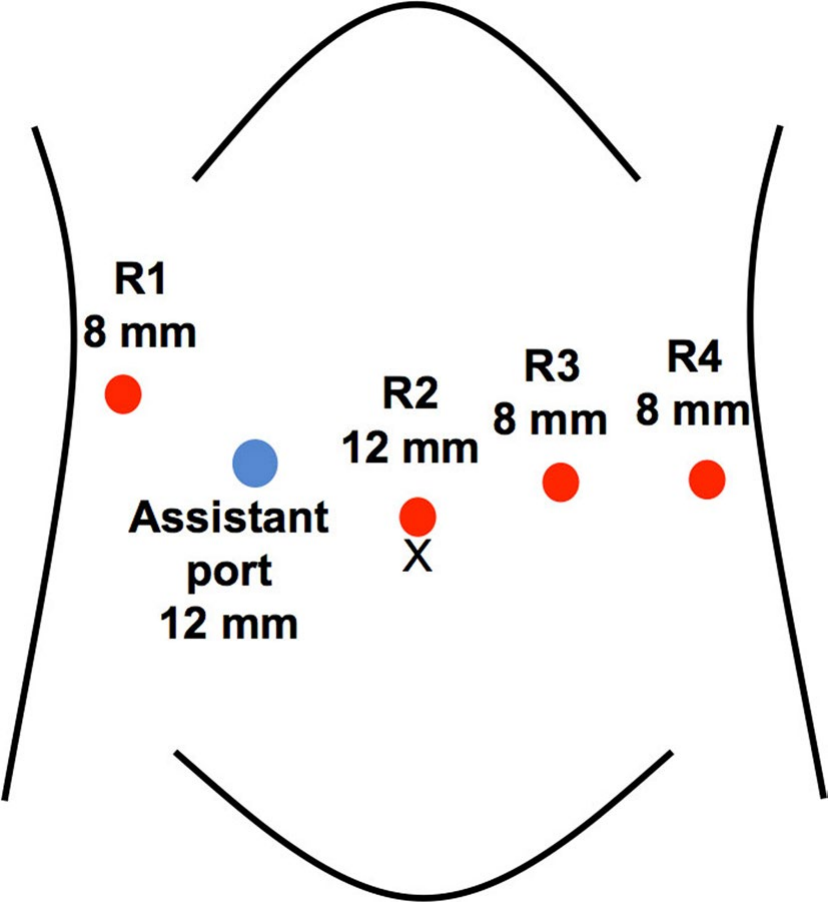
Port placement. The labels, R1–4, indicate the ports for the robotic arms. The size of the R1, R3, and R4 ports was 8 mm. The size of R2 port for the camera scope was 12 mm. An assistant port was placed in the right lateral abdomen

**Fig. 3:**
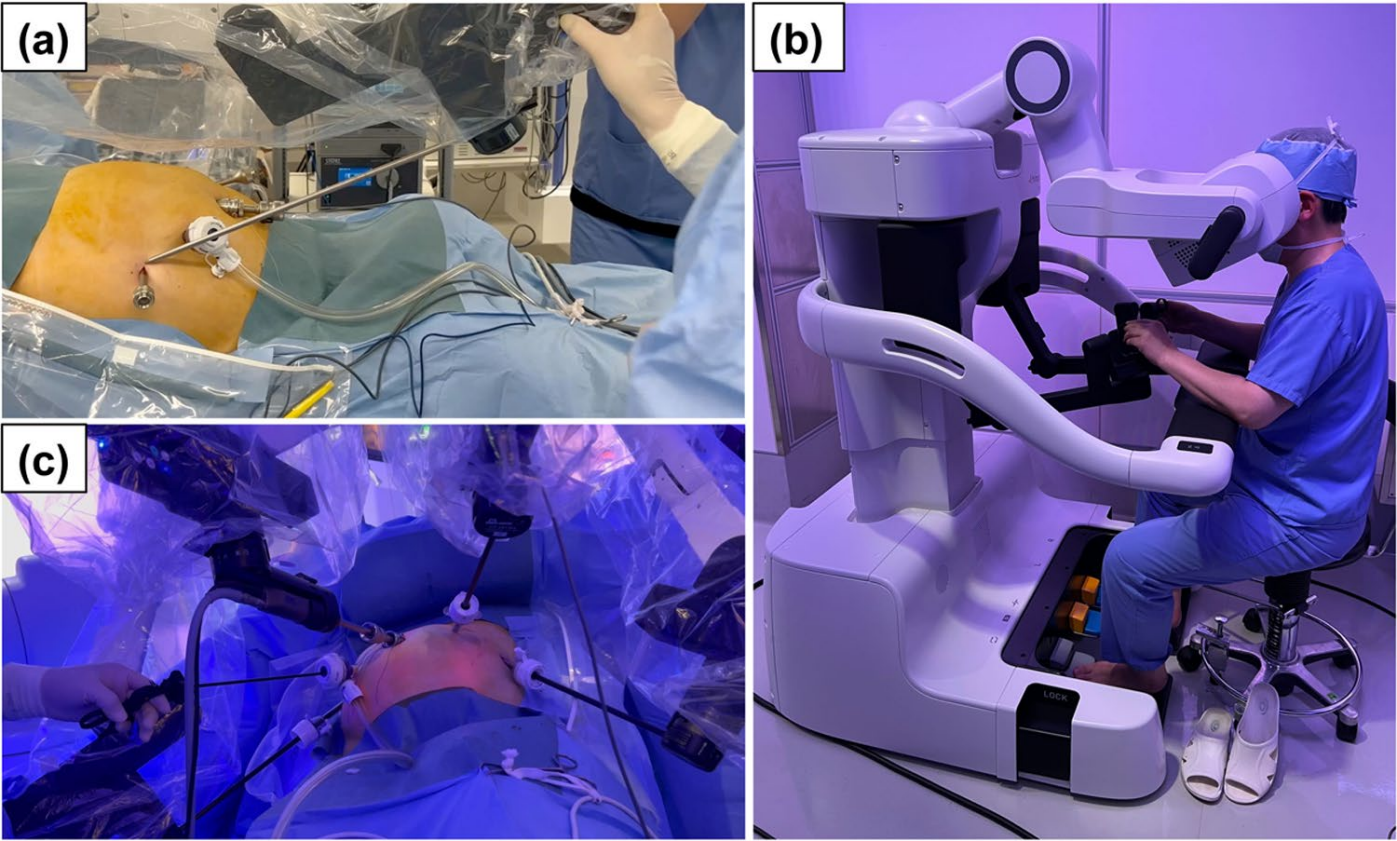
Settings for the hinotori™ Surgical System. **a** The pivot position was set for each robotic arm. **b** The console surgeon was seated in the surgeon cockpit during the surgery. **c** The hinotori™ docking-free system can provide a large space around the trocars

Five trocars, including one port for the assistant surgeon, were placed in the upper abdomen (Fig. 2). The patient was placed in the supine position with the head up at 10° and left side up at 5°. The operating unit was rolled in from the patient’s right side, and the robotic arm base was rotated to parallel to the line connecting the R1 and R4 arms. The arm base was positioned 7.5° upward and 5° left upward, in accordance with the patient’s position. Thereafter, the pivot position was set for each robotic arm because a movement center for each arm on the abdominal wall must be memorized by the robot. Setting the pivot position is specific to the hinotori™ (Fig. 3), and this step is respective to adjustment of a remote center with the da Vinci^®^. However, docking of a robot arm and a corresponding port is unnecessary with the hinotori™ owing to the pivoting step.

**Fig. 4:**
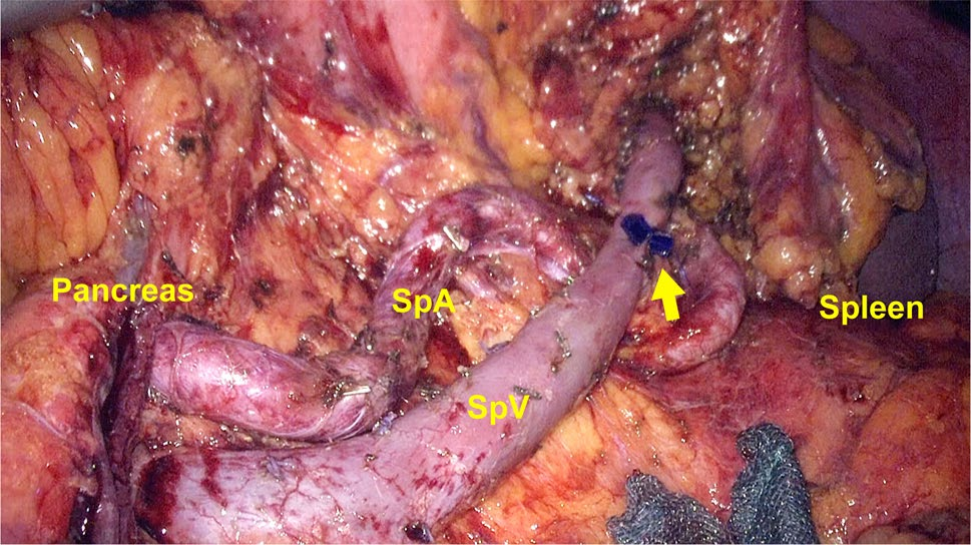
Operative findings. The distal pancreas was resected with conservation of the spleen, splenic artery, and vein. The regional lymph nodes were dissected successfully. The console surgeon closed the injured splenic vein by suturing (arrow). SpA, splenic artery; SpV, splenic vein

The 1st arm is handled by the surgeon’s left hand, the 2nd arm is used for a camera, the 3rd arm is handled by the surgeon’s right hand, and the remaining 4th arm is used for an extra robotic arm that is switched on or off instead of using the 3rd arm. The main instruments used in this surgery were bipolar Maryland forceps for the 1st robotic arm, monopolar curved scissors for the 3rd arm, and universal grasping forceps for the 4th arm. After dividing the greater omentum from the transverse colon and opening the greater and lesser omental sacs, the inferior border of the pancreas was dissected at the isthmus level. The pancreatic body was then mobilized from the retroperitoneum. Following identification of the mesenteric portal venous axis, the avascular plane was dissected between the posterior aspect of the pancreatic parenchyma and the anterior wall of the portal vein. The root of the splenic artery was isolated and retained in place using vascular tape, above the pancreatic body. The pancreatic parenchyma was also isolated and encircled on the portal vein with vascular tape, as for the splenic artery. After sufficient precompression, the pancreas was wrapped with a polyglycolic acid sheet (Neoveil; Gunze Corporation, Tokyo, Japan) and divided using an endoscopic linear stapler that was introduced through the assistant’s port. The splenic vein and splenic artery were carefully isolated from the pancreatic body toward the spleen with dissection of the regional lymph nodes (Fig. 4). The console surgeon ligated or clipped the vessel branches connecting to the pancreas at both the residual and resected sides, and cut the branches using monopolar curved scissors. For some small branches, the residual side was ligated, and the resected side was dissected after precoagulation using bipolar Maryland forceps or dissected with a vessel-sealing device from the assistant port. During this procedure, a small splenic vein was injured, which led to bleeding. The console surgeon closed the injured splenic vein by suturing and avoided splenectomy or conversion to laparotomy. The stump of the pancreatic head was then wrapped with the preserved original omentum by suturing, and a drain was placed around the pancreatic stump. The excised specimen was placed in a plastic specimen bag and extracted through the umbilical port wound, which was enlarged to 5 cm.

The cockpit surgeon performed all procedures using the camera and the robotic arms. The console surgeon directed the assistant to dissect the tissues using a vessel-sealing device, when needed. The hinotori™ is equipped with a clip applier, but the assistant sometimes performed clipping to avoid prolonging the surgical time owing to forceps exchange. No unrecoverable device errors occurred during the operation. The total operation time was 531 min, and the console time was 465 min. The estimated blood loss was 192 ml, and no blood transfusion was required. There was no remarkable subcutaneous emphysema postoperatively.

The postoperative course was uneventful except for a surgical site infection at the drain site, and the patient was treated with oral antibiotic therapy for 7 days. The patient was discharged 19 days after the operation. The pathological diagnosis was a G1 neuroendocrine tumor without regional lymph node metastasis. The patient was prescribed medication for diabetes mellitus, and there has been no tumor recurrence for 6 months after the operation.

## Discussion

The hinotori™ is the first docking-free surgical robot system produced by a Japanese-based company. With this new platform, we expected that more challenging surgery could be performed. We successfully performed robotic spleen-preserving distal pancreatectomy with conservation of the splenic artery and vein for the present patient, which we feel is valuable to report, considering the complicated nature of the procedure.

Prior to this operation, we performed seven spleen-preserving distal pancreatectomies using the da Vinci^®^ Surgical System. The mean operation time was 350 ± 84 min, and the mean blood loss was 101 ± 137 ml. One of the seven patients was diagnosed with postoperative pancreatic fistula grade B in accordance with the definition of the International Study Group of Pancreatic Fistula [11]. No other patients developed complications of Clavien–Dindo classification grade ≥ III [12]. The present case was the second pancreatic surgery with the hinotori™, and it took a long time to determine the pivot position or to exchange the instruments. We consider this to be the reason for the longer operation time with the hinotori™ compared with the da Vinci®. We believe that once we become fully familiar with the pivot position and the instruments exchange procedures, we can shorten the operation time. The blood loss was higher in the present case compared with prior cases using the da Vinci^®^ because of the injury to the splenic vein. However, the console surgeon was able to close the vessel by suturing and avoid splenectomy or conversion to laparotomy. The hinotori™ performed sufficiently as a surgical robot system in highly advanced pancreatic surgery; however, currently, we cannot say that this system is superior to the da Vinci®.

Both the hinotori™ and the da Vinci^®^ systems have articulated arms, and the movement is meticulous and precise, with tremor filtering and motion scaling. Both systems have four arms, and the console surgeon can perform the operative procedures similarly with either system. However, the hinotori™ has features that differ from the da Vinci^®^ (Table 1). The degrees of freedom for each arm are greater with the hinotori™ compared with the da Vinci^®^, which increases the flexibility of the robotic arm movements with the hinotori™. However, the hinotori™ has no adjustability of the axes. Additionally, da Vinci^®^ has a patient clearance feature to avoid collision with the robotic arms or the patient’s body. Therefore, the patient-side surgeons can resolve arm interference without interrupting the surgical procedure of the console surgeon. With the hinotori™, the console surgeon must stop the procedure and adjust the arm position in accordance with the instruction of the patient-side surgeons. Regardless of the surgical robot system, we must pay attention to both interference between the robotic arms and collision between the robotic arm and the patient’s body at all times, during the operation. Additionally, when the axes are fully extended, robotic arm movement is greatly restricted with the hinotori™. Compared with the da Vinci^®^, the hinotori™ has a disadvantage during surgical procedures in the ventral abdomen or the horizontally opposite side of the lateral arms because of the full extension of the arms. We must determine the port placement carefully with the hinotori™ to prevent extension of the robotic arms.

**Table 1:** Comparison of the characteristics of the hinotori™ and da Vinci® robotic surgical systems

	hinotori™ surgical system	The da Vinci^®^ surgical system
Manufacturer	Medicaroid Corporation, Kobe, Japan	Intuitive Surgical, Inc., Sunnyvale, CA, USA
Regulatory approval (year)	Japanese Ministry of Health, Labor and Welfare (2020)	US Food and Drug Administration (1998)
Camera	Three-dimensional high-definition	Three-dimensional high-definition
Motion scaling	Yes	Yes
Tremor filtering	Yes	Yes
Number of robotic arms	4	4
Number of axes	8	7
Adjustability of the axes	No	Yes, with a patient clearance button
Docking	Docking-free design	Docking design
Center of motion	Pivot position determined by software	Remote center determined by the dedicated trocar
Number of instrument types	11	39

The greatest difference between the systems is that the hinotori™ system software determines the pivot position for each arm without attaching the trocar. This docking-free system can provide a large space around the trocars, as shown in Fig. 3c. This large space and flexible movement can reduce interference between the robotic arms. Notably, the hinotori™ might be more appropriate for Japanese patients, whose bodies are usually smaller than those of Western patients. Moreover, this docking-free design is expected to reduce subcutaneous emphysema caused by abdominal wall tissue damage due to excessive traction. Although the patient in the present case did not develop postoperative subcutaneous emphysema, additional experience is required with the hinotori™ regarding this issue. Nevertheless, we emphasize that the hinotori™ has no dedicated trocars, and the surgeon can freely select the type of trocar. The surgeons can exchange or replace the trocars more easily and smoothly compared with the da Vinci^®^. In contrast, pivoting is necessary with the hinotori™ because of this docking-free system instead of adjustment of a remote center, as with the da Vinci^®^. It usually took longer before starting actual procedures in the surgeon cockpit with the hinotori™ compared with the da Vinci^®^, as seen in the present case. From this experience, we consider it is necessary for the surgical team, including the assistant surgeons, operating nurses, and staff, to practice the setting of the hinotori™ before the operation. Pivoting and exchanging the instruments can easily lead to lost time.

The number of instrument types with the hinotori™ is much lower than that for the da Vinci^®^. In particular, a dedicated vessel-sealing device has not yet been developed for the hinotori™; therefore, the console surgeon must use a limited number of devices. The development of additional types of forceps is desired in the future.

With the hinotori™, the console surgeon feels as if the forceps are floating because of the slight delay between the surgeon’s console and the surgical robotic movement. Additionally, excessive safety measures to prevent arm collisions with the hinotori™ sometimes result in a limited range of motion of the arms, as reported by Miyo et al. [13]. Medicaroid Corporation is currently updating the software in the hinotori™ to satisfy surgeons’ demands. As a result, the sense of floating with the forceps and the excessive safety measures have gradually improved. Currently, the first-generation hinotori™ may have some inferiority compared with the da Vinci^®^; however, the next generation of this system is expected to have better functions.

## Conclusions

We successfully performed highly advanced spleen-preserving distal pancreatectomy with conservation of the splenic artery and vein, using the new hinotori™ Japanese domestic surgical robot platform. We expect that various types of surgical robots will be developed in the future. Therefore, it is important to be familiar with the features of each robot to establish a surgical strategy for patients with various conditions.

## Data Availability

All data supporting the conclusions of this article are included within the published article.
